# Characterization of tick organic anion transporting polypeptides (OATPs) upon bacterial and viral infections

**DOI:** 10.1186/s13071-018-3160-6

**Published:** 2018-11-14

**Authors:** Vikas Taank, Wenshuo Zhou, Xuran Zhuang, John F. Anderson, Utpal Pal, Hameeda Sultana, Girish Neelakanta

**Affiliations:** 1 Department of Biological Sciences, Old Dominion University, Norfolk, VA USA; 2 Department of Veterinary Medicine, Virginia-Maryland Regional College of Veterinary Medicine, University of Maryland, College Park, MD USA; 3 Department of Entomology, Connecticut Agricultural Experiment Station, New Haven, CT USA; 4 Center for Molecular Medicine, College of Sciences, Old Dominion University, Norfolk, VA USA

**Keywords:** Organic anion transporting polypeptide, Kynurenine aminotransferase, *Borrelia burgdorferi*, Langat virus, *Ixodes scapularis*, *Anaplasma phagocytophilum*, Xanthurenic acid, Anti-vector vaccine

## Abstract

**Background:**

*Ixodes scapularis* organic anion transporting polypeptides (OATPs) play important roles in tick-rickettsial pathogen interactions. In this report, we characterized the role of these conserved molecules in ticks infected with either Lyme disease agent *Borrelia burgdorferi* or tick-borne Langat virus (LGTV), a pathogen closely related to tick-borne encephalitis virus (TBEV).

**Results:**

Quantitative real-time polymerase chain reaction analysis revealed no significant changes in *oatps* gene expression upon infection with *B. burgdorferi* in unfed ticks. Synchronous infection of unfed nymphal ticks with LGTV *in vitro* revealed no significant changes in *oatps* gene expression. However, expression of specific *oatps* was significantly downregulated upon LGTV infection of tick cells *in vitro*. Treatment of tick cells with OATP inhibitor significantly reduced LGTV loads, kynurenine amino transferase (*kat*), a gene involved in the production of tryptophan metabolite xanthurenic acid (XA), levels and expression of several *oatp*s in tick cells. Furthermore, bioinformatics characterization of OATPs from some of the medically important vectors including ticks, mosquitoes and lice revealed the presence of several glycosylation, phosphorylation and myristoylation sites.

**Conclusions:**

This study provides additional evidence on the role of arthropod OATPs in vector-intracellular pathogen interactions.

**Electronic supplementary material:**

The online version of this article (10.1186/s13071-018-3160-6) contains supplementary material, which is available to authorized users.

## Background

Blood-feeding arthropods such as ticks, mosquitoes and lice are important vectors for several human pathogens [1]. Based on the disability adjusted life year (DALY) estimates, one sixth of the human population worldwide are prone to infections caused by pathogens transmitted by arthropods, including ticks, mosquitoes and lice [1]. These arthropods have evolved different blood-feeding behaviors [2–4]. The control strategies for these arthropods are limiting, as use of several acaricides have shown to be ineffective in many instances [5]. Recent progress in new methods such as the development of transmission-blocking vaccines targeting conserved proteins across various arthropod species has provided a significant promise for the treatment or control of the diseases transmitted by these arthropods [6–9].

The presence of the multigene family of organic anion transporting polypeptides (OATPs) across various arthropods and vertebrates suggests that it has an important role in various aspects of the physiology of these organisms [10–12]. OATPs in humans are now recognized as determinants of the transmembrane passage of drugs that are important for pharmacokinetics [13]. Human OATPs localize to barrier epithelial cells that facilitate the uptake of several substances including toxins, hormones, metabolites and those involved in cell signaling [10, 11, 13, 14]. The N-terminus and C-terminus parts of vertebrate OATPs are noted to be intracellular [10, 11, 13–15]. However, the remaining part of OATPs is organized into several transmembrane domains linked by short intracellular loops and extracellular loops that face outside the membrane [10, 11, 13–15].

The black-legged *Ixodes scapularis* tick is a medically important vector for various human pathogens including *Borrelia burgdorferi*, *Anaplasma phagocytophilum*, *Babesia microti*, *Ehrlichia muris*-like agent (EMLA), Powassan virus (POWV) and *Borrelia miyamotoi* [16–19]. Other *Ixodes* species transmit tick-borne encephalitis virus (TBEV) [20]. Langat virus (LGTV), a close member of TBEV, is considered as a model biosafety level 2 pathogen to study infection dynamics of TBEV [20, 21]. LGTV readily infects *I. scapularis* ticks and the ISE6 tick cell line derived from these ticks [21, 22]. A recent study reported the presence of nine OATPs in *I. scapularis* ticks [23]. Phylogenetic analysis of *I. scapularis* and other tick OATPs revealed that these OATPs are clustered with various other orthologs from medically important blood-feeding arthropods such as lice and mosquitoes [23].

We recently reported the importance of specific OATPs in *I. scapularis* tick-rickettsial pathogen interactions [12]. We have shown that *A. phagocytophilum* specifically modulates tick OATP, *isoatp4056*, and kynurenine amino transferase (*kat*), a gene involved in the production of tryptophan metabolite xanthurenic acid (XA), for its survival in ticks. RNAi mediated analysis revealed that knockdown of *isoatp4056* and/or *kat*, affected *A. phagocytophilum* survival in ticks. Our results noted interesting cross-talk between tick OATP and XA in the survival of this rickettsial pathogen in its vector host. Arthropod XA mediates malarial parasite gametogenesis in mosquitoes [24, 25]. Collectively, these studies suggest that in addition to their role in physiological processes in various organisms, OATPs also participate in vector-pathogen interactions. The role of OATPs in the interactions of ticks with other pathogens remains to be explored.

In view of the development of anti-vector vaccines as an effective means to target various arthropods, understanding the role of conserved protein families such as OATPs in vector biology and interactions with different pathogens remains important. In this study, we performed molecular analysis of OATPs in the interactions of ticks with an extracellular bacterial pathogen and an intracellular tick-borne virus.

## Methods

### Bacterial/viral isolates, ticks and tick cell line

*Borrelia burgdorferi* strain B31-A3 or Langat virus (LGTV) strain (LGT-TP21) was used throughout this study. These strains will be herein referred to as *B. burgdorferi* and LGTV, respectively. *Borrelia burgdorferi*-infected ticks were generated as described [26] and RNA extractions from ticks were performed in the laboratory of Dr Utpal Pal at the University of Maryland, USA. Uninfected nymphs used in generating LGTV-infected ticks were obtained from a tick colony at the Connecticut Agricultural Experiment Station, New Haven, CT, USA. The ISE6 tick cell line was provided by Dr Ulrike Munderloh at the University of Minnesota (St Paul, MN, USA) and maintained as described [12]. Both uninfected and LGTV-infected ticks used in this study were maintained in an environmental chamber (Parameter Generation and Control, Black Mountain, NC, USA) set at a temperature of 23 ± 1 °C, 95% humidity and a 14/10 h light/dark photoperiod regiment.

### *In vitro* generation of LGTV-infected ticks

Synchronous LGTV infection in uninfected ticks was performed as described [21]. Briefly, about 25 nymphal ticks were immersed in 0.5 ml of complete Dulbecco’s modified Eagle’s medium (DMEM) containing 1 × 10^7^ pfu/ml of LGTV viral stock and incubated at 34 °C for 1 h. Vials containing ticks in the medium were vortexed every 10 min to redistribute the medium over ticks. After incubation, ticks were thoroughly washed five times with 1× phosphate-buffered saline (PBS) and incubated for 17 days in an environmental chamber. RNA from these ticks were extracted and tested for the presence of both positive and negative sense RNA strands of LGTV as described [21, 27].

### RNA extraction and quantitative real-time PCR (QRT-PCR) analysis

Total RNA from uninfected or LGTV-infected nymphs and tick cells was generated using an Aurum Total RNA mini kit (Bio-Rad, Hercules, USA) following the manufacturer’s instructions. The cDNA was later synthesized using an iScript cDNA synthesis kit (Bio-Rad) and used for QRT-PCR reactions [12, 27]. The amount of tick *beta-actin* transcripts was used to normalize the amount of template in each reaction. QRT-PCR assays were performed using iQ-SYBR Green Supermix (Bio-Rad) and a Bio-Rad CFX96 QPCR machine. *Borrelia burgdorferi flaB* gene transcripts or LGTV positive or negative RNA strands were quantified in the cDNA samples. Serial dilutions (10-fold) ranging from 1 to 0.00001 ng of respective fragments were used to generate a standard curve. QRT-PCR oligonucleotides for *oatps*, actin, *kat*, *B. burgdorferi flaB* and for the detection of LGTV are published in our previous studies [12, 27, 28].

### LGTV infection of tick cells

LGTV infection of tick cells was performed as described [27]. All infection experiments in tick cells were performed with 1 MOI of LGTV. 1 × 10^5^ tick cells were seeded in L-15B300 medium onto 12-well plates and incubated for 24 h followed by infection with 1 MOI of LGTV and collection of cells at 24 and 72 h post-infection (pi). RNA from these collected cells was extracted and processed for cDNA synthesis and QRT-PCR analysis.

### Tick cell line experiments with OATP inhibitor

Inhibitor treatment was performed as described [12]. 1 × 10^5^ tick cells were plated in 12-well cell culture plates and incubated for 20 h. After incubation, 100 μM of ±-sulfinpyrazone (SPZ, purchased from Santa Cruz Biotechnology Inc., Dallas, USA) was added and cells were incubated for an additional 4 h followed by LGTV infection. 0.5 N NaOH was used to prepare 10 mM SPZ stocks. For experiments, a one-tenth diluted (with 1× PBS) solution (final concentration 1mM SPZ) was used. A mock solution was prepared in a similar way but without SPZ. An equal volume of mock solution (corresponding to 100 μM volume of SPZ) was added to control cell culture wells. After 24 h pi, cells were processed for RNA extractions followed by cDNA synthesis and QRT-PCR analysis to measure *oatp* or *kat* transcripts and LGTV loads.

### Bioinformatic analysis

The amino acid sequences that contained OATP signature sequence WxGxWWxG were downloaded from GenBank and individually analyzed at PROSITE (http://prosite.expasy.org/) as described [29–31]. Biology WorkBench (San Diego Supercomputer Center) at http://workbench.sdsc.edu/ and the National Center for Biotechnology Information conserved domain search (NCBI-CD) at http://www.ncbi.nlm.nih.gov/Structure/cdd/wrpsb.cgi were used for the prediction of glycosylation, myristoylation, protein kinase C phosphorylation, casein kinase II phosphorylation, tyrosine phosphorylation, cAMP-dependent protein kinase phosphorylation and identification of Kazal domain sites. For data analysis, the post-translational modification sites that are present either outside or inside regions of OATPs but not on the transmembrane regions were considered. TMHMM http://www.cbs.dtu.dk/services/TMHMM/ server v.2.0 (prediction of transmembrane helices in proteins) was used to predict transmembrane and outside or inside regions of OATP. For Kazal site prediction, full-length OATP sequences were considered.

### GenBank accession numbers

The GenBank accession numbers for the OATP sequences used in the study are as follows: *D. melanogaster* (AAF46824-AAF46826, AAF49332, NP_609055, NP_001260417), *Aedes aegypti* (XP_001658583, XP_001659726, XP_001660406, XP_001660407, XP_001661188), *Anopheles gambiae* (XP_314819, XP_316669, XP_319187, XP_557860, XP_001237849), *Culex quinquefasciatus* (EDS26845, EDS34303, EDS45569, EDS45572), *Pediculus humanus corporis* (EEB11548, EEB18131, EEB20444, EEB20468), *Rhipicephalus pulchellus* (JAA58190, JAA59396, JAA59849, JAA64227), *Amblyomma americanum* (ACH98103) and *Ixodes scapularis* (DAA34891, XP_002400770, XP_002412161, XP_002414101, XP_002434179, XP_002404592, XP_002404594, XP_002415171, XP_002435666).

### Statistics

A non-paired two-tailed Student’s t-test and nonparametric Mann-Whitney tests from GraphPad Prism6 software and Microsoft Excel 2016 were used to calculate statistical significance. Graphs were generated using GraphPad Prism6 software. Horizontal lines in the graphs represent the mean values. *P* < 0.05 was considered significant and shown at relevant places.

## Results

### *Borrelia burgdorferi* has no impact on the expression of any of the *I. scapularis oatps* in unfed ticks

**Fig. 1:**
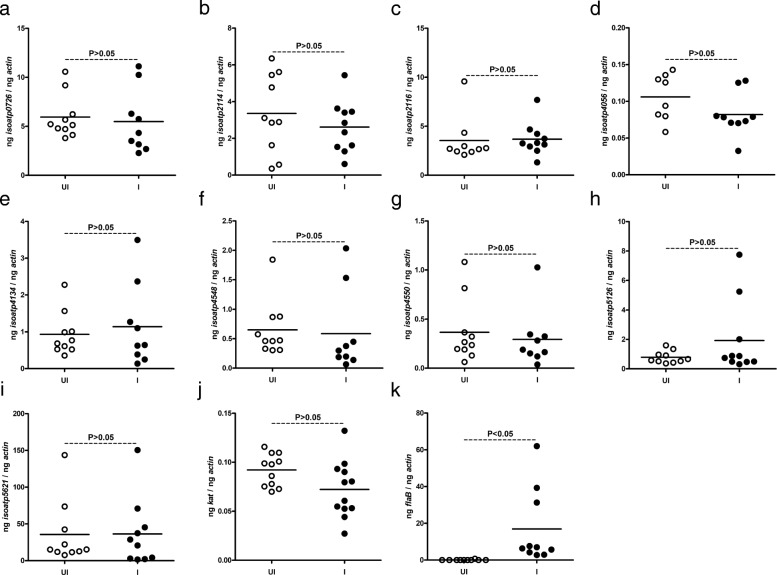
*B. burgdorferi* has no impact on the expression of nine *I. scapularis oatps* in unfed nymphal ticks. Results from QRT-PCR assays are shown in all panels. **a-i** Expression of nine *I. scapularis* OATPs: *isoatp*-*0726* (**a**), -*2114* (**b**), -*2116* (**c**), -*4056* (**d**), -*4134* (**e**), -*4548* (**f**), -*4550* (**g**), -*5126* (**h**) and -*5621* (**i**) in unfed uninfected or *B. burgdorferi*-infected ticks. Levels of tick *kat* (**j**) or spirochete *flaB* (**k**) mRNA in *B. burgdorferi*-infected unfed nymphal ticks are also shown. Uninfected ticks were used as controls in all panels. In all panels, open circles represent data from uninfected (UI) and closed circles represent data from infected (I) ticks. Each circle represents data from one tick. The amount of mRNA levels of *flaB* or *oatps* or *kat* was normalized to the amount of tick beta-actin mRNA levels. The *P*-values indicate the results from statistical analyses

Previous studies have reported expression of nine *oatps* in unfed *I. scapularis* ticks [12, 23]. Our recent study reported upregulation of specific *oatps* and *kat* upon *A. phagocytophilum* infection in unfed ticks [12]. To test whether similar observation was evident upon infection with an extracellular pathogen, we used *B. burgdorferi*-infected unfed ticks*.* We found no significant (*P* > 0.05) differences (Additional file 1: Table S1) in the expression levels of *isoatp-0726* (Fig. 1a), -*2114* (Fig. 1b), -*2116* (Fig. 1c), -*4056* (Fig. 1d), -*4134* (Fig. 1e), -*4548* (Fig. 1f), -*4550* (Fig. 1g), -*5126* (Fig. 1h) and -*5621* (Fig. 1i) between unfed uninfected and *B. burgdorferi*-infected ticks. In addition, no significant (*P* > 0.05) difference (Additional file 1: Table S1) in *kat* gene expression was noted between unfed uninfected and *B. burgdorferi*-infected ticks (Fig. 1j). As expected, QRT-PCR analysis revealed presence of *flaB* transcripts only in *B. burgdorferi*-infected ticks but not in uninfected ticks (Fig. 1k). These results show that unlike the intracellular pathogen *A. phagocytophilum* (as reported in our previous study [12]), the extracellular pathogen *B. burgdorferi* has no impact on the expression of any arthropod *oatps* and *kat* gene in unfed ticks.

### Synchronous infection of LGTV does not impact OATP expression in unfed ticks

**Fig. 2:**
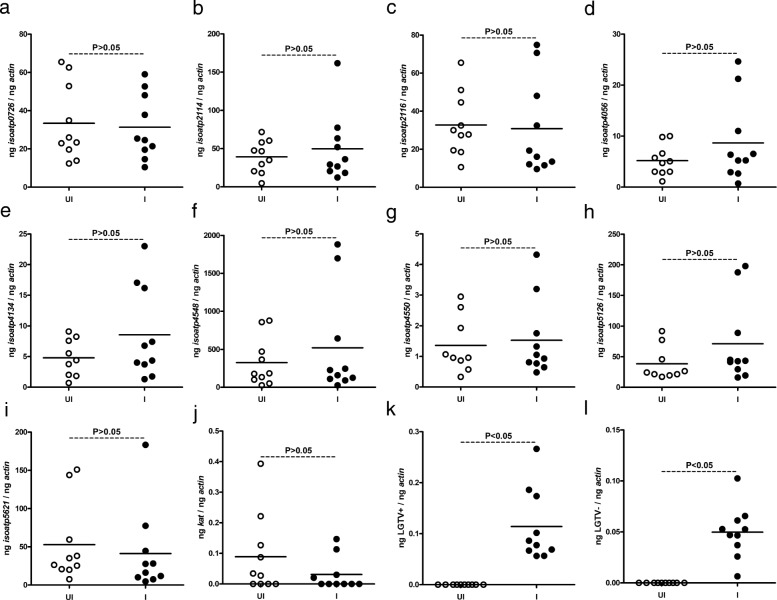
LGTV has no impact on the expression of nine *I. scapularis oatps* in synchronously-infected unfed nymphal ticks. Data from QRT-PCR assays are shown in all panels. **a-i** Expression of nine *I. scapularis* OATPs: *isoatp*-*0726* (**a**), -*2114* (**b**), -*2116* (**c**), -*4056* (**d**), -*4134* (**e**), -*4548* (**f**), -*4550* (**g**), -*5126* (**h**) and -*5621* (**i**) in unfed uninfected or LGTV-synchronously-infected ticks is shown. **j** Levels of tick *kat* mRNA in unfed uninfected or LGTV-synchronously-infected ticks. Levels of viral positive-sense strand (**k**) or negative sense strand (**l**) in LGTV-synchronously-infected ticks are shown. Uninfected ticks were used as controls in all panels. In all panels, open circles represent data from uninfected (UI) and closed circles represent data from LGTV synchronously-infected (I) ticks. Each circle represents data from one tick. The amount of mRNA levels of LGTV positive- or negative-sense strands or *oatps* or *kat* was normalized to the amount of tick beta-actin mRNA levels. The *P*-values indicate the results from statistical analyses

LGTV readily infects *I. scapularis* ticks [21]. We generated synchronously infected LGTV ticks *in vitro* (as described in Methods) to test whether tick-borne viruses have any impact on *oatp* expression. We found no significant (*P* > 0.05) differences (Additional file 1: Table S1) in the expression levels of *isoatp*-*0726* (Fig. 2a), -*2114* (Fig. 2b), -*2116* (Fig. 2c), -*4056* (Fig. 2d), -*4134* (Fig. 2e), -*4548* (Fig. 2f), -*4550* (Fig. 2g), -*5126* (Fig. 2h) and -*5621* (Fig. 2i) between unfed uninfected ticks or LGTV-infected ticks. In addition, no significant (*P* > 0.05) difference (Additional file 1: Table S1) in *kat* gene expression was noted between unfed uninfected or LGTV-infected ticks (Fig. 2j). QRT-PCR analysis revealed presence of both positive (Fig. 2k) and negative (Fig. 2l) strands of virus in LGTV-infected ticks. As expected, no LGTV RNA was detected in uninfected controls (Fig. 2k, l). These results show that LGTV does not impact *oatp* and *kat* expression in unfed ticks.

### Expression of specific *I. scapularis oatp* transcripts was downregulated upon LGTV infection of tick cells

**Fig. 3:**
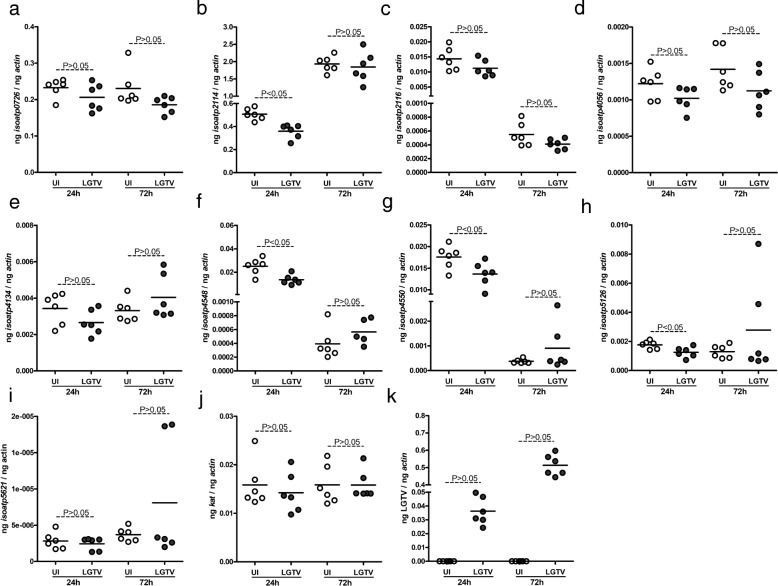
LGTV infection of tick cells *in vitro* affects expression of *isoatp2114*, *isoatp4548*, *isoatp4550* and *isoatp5126*. Data from QRT-PCR assays are shown in all panels. **a-i** Expression of nine *I. scapularis* OATPs: *isoatp*-*0726* (**a**), -*2114* (**b**), -*2116* (**c**), -*4056* (**d**), -*4134* (**e**), -*4548* (**f**), -*4550* (**g**), -*5126* (**h**), -*5621* (**i**) and *kat* mRNA (**j**) in unfed uninfected ticks and LGTV-infected tick cells at 24 and 72 h pi. **k** Levels of viral RNA in LGTV-infected ISE6 tick cell line (LGTV) at 24 and 72 h pi. is shown. Uninfected (UI) cells were used as controls in all panels. In all panels, open circles represent data from uninfected (UI) and closed circles represent data from LGTV-infected (LGTV) tick cells. Each circle represents data from one cell culture well. LGTV loads or *oatps* or *kat* mRNA levels was normalized to the amount of tick beta-actin mRNA levels. The *P*-values indicate the results from statistical analyses

We next tested whether LGTV infection has any impact on the *oatp* mRNA levels in tick cells. We selected 24 and 72 h pi as early and late infection stages, respectively, and expression of all nine *oatp* genes were analyzed (Fig. 3). No significant (*P* > 0.05) differences (Additional file 1: Table S1) in the expression levels of *isoatp*-*0726* (Fig. 3a), -*2116* (Fig. 3c), -*4056* (Fig. 3d), -*4134* (Fig. 3e) and -*5621* (Fig. 3i) between uninfected and LGTV-infected tick cells at 24 h pi were noted. However, significant downregulation of *isoatps*-*2114* (Fig. 3b), *-4548* (Fig. 3f), *-4550* (Fig. 3g) and *-5126* (Fig. 3h) was evident in LGTV-infected tick cells in comparison to uninfected control at 24 h pi. No significant (*P* > 0.05) differences (Additional file 1: Table S1) in the expression of all nine *oatps* were noted between LGTV-infected tick cells and uninfected controls at 72 h pi. In addition, no significant (*P* > 0.05) difference (Additional file 1: Table S1) in *kat* gene expression was noted between unfed uninfected and LGTV-infected tick cells (Fig. 3j). LGTV readily infected tick cells with increased viral loads at 72 h pi in comparison to 24 h pi (Fig. 3k). No morphological difference was evident between uninfected or LGTV-infected tick cells at both time points (Additional file 1: Figure S1). These results suggest that specific *oatp* genes are modulated at early but not at later stages of LGTV infection of tick cells.

### Inhibition of OATP affects LGTV burden in tick cells

**Fig. 4:**
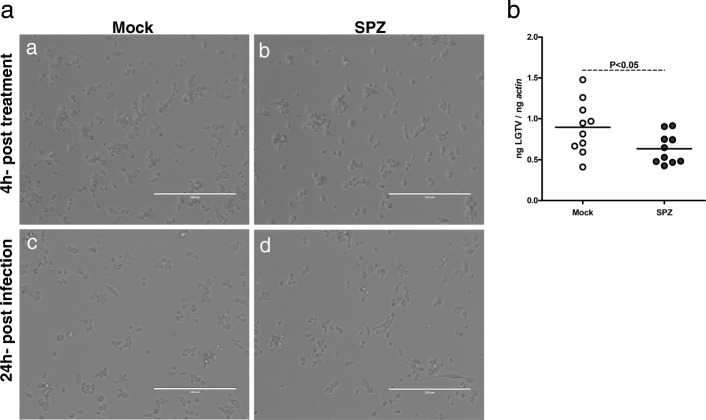
Inhibition of OATPs affect LGTV loads in tick cells. **a** Representative phase-contrast images of LGTV-infected mock-treated or SPZ-treated tick cells (4 h post-treatment, before infection) followed by 24 h post-LGTV-infection. **b** QRT-PCR analysis showing levels of viral RNA in LGTV-infected mock- or SPZ-treated tick cells at 24 h pi. Open circles represent data from LGTV-infected mock-treated and closed circles represent data from SPZ-treated tick cells at 24 h pi. Each circle represents data from one cell culture well. LGTV loads were normalized to the amount of tick beta-actin levels. In **b**, the *P*-value indicates the result from statistical analysis. *Scale-bars*: 200 μm

The downregulation of specific *oatps* suggests an important role for these molecules in early tick-LGTV interactions. We tested whether treatment of tick cells with SPZ, a general inhibitor of OATP, has any impact on LGTV infection at early stages. Tick cells were treated with 100 μM of SPZ as described [12], followed by LGTV infection for 24 h. Microscopic observations revealed no morphological differences in tick cells that were either mock-treated or SPZ-treated at 4 h post-treatment followed by LGTV-infection for 24 h (Fig. 4a). In addition, no cytotoxicity was observed upon treatment of uninfected tick cells with 100 μM of SPZ at 4, 24, 48 and 72 h post-treatment (Additional file 1: Figure S2). However, significantly (*P* < 0.05) reduced viral loads were evident in SPZ-treated LGTV-infected tick cells in comparison to mock-treated control at 24 h pi (Fig. 4b, Additional file 1: Table S1). These results suggest that OATPs play important roles in the survival of LGTV in tick cells.

### Treatment of LGTV-infected tick cells with OATP inhibitor affects expression of several *oatps* and *kat* gene expression

**Fig. 5:**
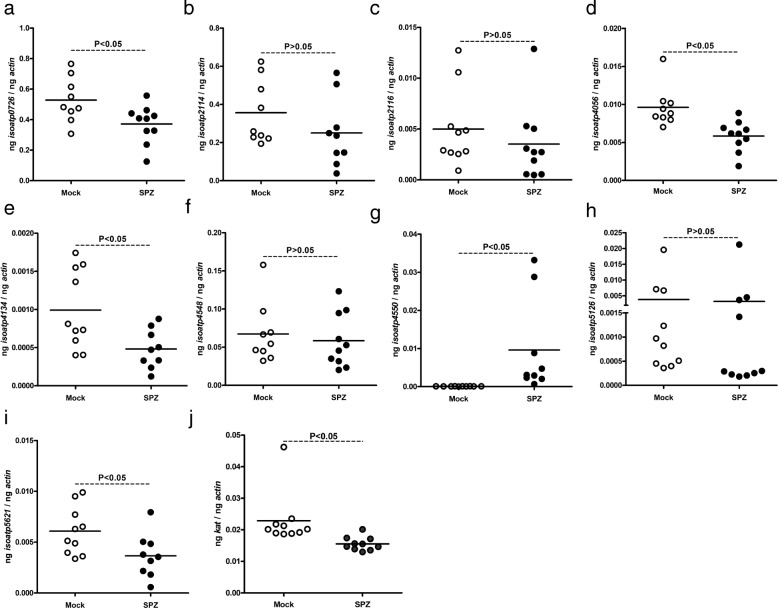
Treatment of tick cells with OATP inhibitor affects *oatps* and *kat* gene expression. Data from QRT-PCR assays are shown in all panels. Expression of nine *I. scapularis* OATPs: *isoatps*-*0726* (**a**), -*2114* (**b**), -*2116* (**c**), -*4056* (**d**), -*4134* (**e**), -*4548* (**f**), -*4550* (**g**), -*5126* (**h**), -*5621* (**i**) and *kat* (**j**) mRNA levels in LGTV-infected mock- or OATP-inhibitor (SPZ)-treated tick cells at 24 h pi. In all panels, open circles or closed circles represent data from LGTV-infected mock- or SPZ-treated tick cells at 24 h pi, respectively. Each circle represents data from one cell culture well. The *oatps* and *kat* mRNA levels were normalized to the tick beta-actin mRNA levels. The *P*-values indicate the results from statistical analyses

We then assessed whether treatment of tick cells with OATP inhibitor has any effect on the *oatps* mRNA levels (Fig. 5, Additional file 1: Table S1). QRT-PCR analysis revealed significant (*P* < 0.05) downregulation of *isoatps*-*0726* (Fig. 5a), *-4056* (Fig. 5d), *-4134* (Fig. 5e) and *-5621* (Fig. 5i) in SPZ-treated LGTV-infected tick cells in comparison to mock-treated control at 24 h pi. In addition, we noted a significant (*P* < 0.05) upregulation of *isoatp4550* in SPZ-treated LGTV-infected tick cells in comparison to mock-treated control at 24 h pi (Fig. 5g). No significant (*P* > 0.05) differences in the expression levels of *isoatps*-*2114* (Fig. 5b), -*2116* (Fig. 5c), -*4548* (Fig. 5f), -*5126* (Fig. 5h) was noted between SPZ-treated LGTV-infected tick cells in comparison to the mock-treated control at 24 h pi. Our previous study suggests KAT as an upstream molecular player that impacts *isoatp4056* expression [12]. QRT-PCR analysis revealed a significant (*P* < 0.05) reduction in *kat* transcripts (Additional file 1: Table S1) in SPZ-treated LGTV-infected tick cells in comparison to the mock-treated control at 24 h pi (Fig. 5j). These results clearly suggest an interplay among OATPs in the early stages of tick-LGTV interactions.

### Bioinformatic analysis of OATPs from ticks, mosquitoes and lice

**Fig. 6:**
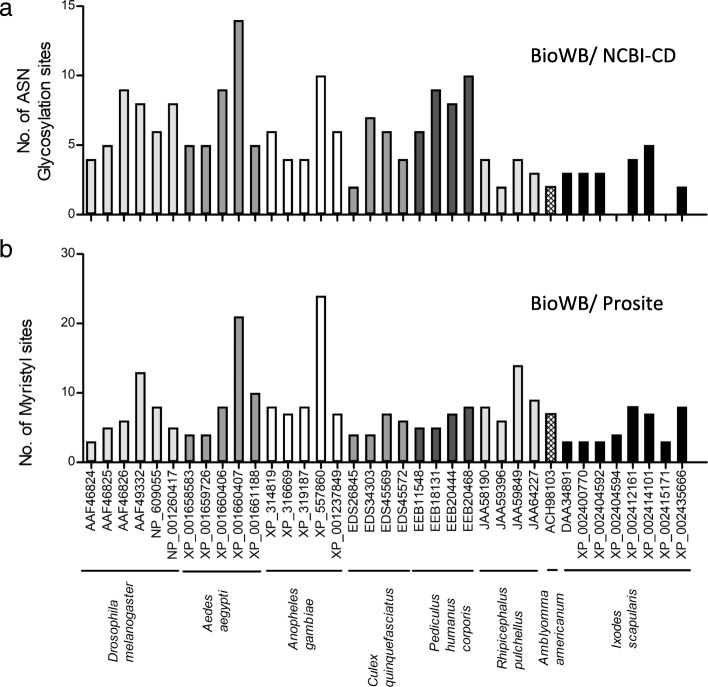
Analysis of glycosylation and myristoylation sites in OATPs from medically important vectors. Amino acid sequences of several OATPs were individually analyzed at Biology WorkBench (**a**, **b**), NCBI conserved domain search (**a**) and PROSITE (**b**) databases for glycosylation (**a**) and myristoylation (**b**) sites. Histograms represent the number of post-translational modification sites on each OATP. The post-translational modifications in the transmembrane regions within each OATP were not considered for histogram plots. GenBank accession numbers and organism names are shown at the bottom of the figure

Post-translational modification is a key strategy that pathogens use to modulate functions of host factors that play central roles in cell signaling [32]. Our previous findings on the role of the OATP-KAT pathway in rickettsial pathogen-tick interactions [12] and the present findings on their roles in LGTV-tick interactions suggest that these conserved molecules play central roles in vector-pathogen interactions. Post-translational modifications are critical for OATPs to function efficiently [33–35]. Therefore, understanding putative post-translational modification sites on OATPs is highly required. As OATPs are shown to be present in many arthropod species [23, 36–38], we performed a comparative analysis of *I. scapularis* OATPs with orthologs from other medically important vectors and the fruit fly *Drosophila melanogaster*. A previous study reported the phylogenetic analysis of OATPs from different species that revealed *I. scapularis* OATPs are segregated with other tick OATPs [23]. In this study, the primary amino acid sequences (that contained OATP signature sequence, WxGxWWxG) of eight *I. scapularis* OATPs, five *Aedes aegypti* OATPs, five *Anopheles gambiae* OATPs, four *Culex quinquefasciatus* OATPs, four *Pediculus humanus corporis* OATPs, four *Rhipicephalus pulchellus* OATPs, one *Amblyomma americanum* OATP and six *D. melanogaster* OATPs were downloaded from the National Center for Biotechnology Information (NCBI) database. Variable length of amino acid sequences among different tick OATPs was noticed (Additional file 1: Tables S2 and S3). Each OATP amino acid sequence was first individually analyzed at TMHMM server v.2.0 (prediction of transmembrane helices in proteins) followed by selection of regions that are exposed outside the plasma membrane and regions that are inside the plasma membrane (Additional file 1: Tables S2 and S3). The transmembrane regions within each OATP sequence were excluded from the analysis. The regions that are outside and inside the plasma membrane were considered to predict conserved and/or unique post-translational modifications in the sequences. Based on the analysis, all but two OATP sequences were predicted to carry at least two N-glycosylation sites (Fig. 6a and Additional file 1: Table S2). OATP from *Ae. aegypti* (GenBank: XP_001660407) was predicted to carry the highest number (14 sites), while, *I. scapularis* (GenBank: XP_002435666), *A. americanum* (GenBank: ACH98103), *R. pulchellus* (GenBank: JAA59396) and *Cx. quinquefasciatus* (GenBank: EDS26845) were predicted to carry the lowest (2 sites) number of N-glycosylation sites (Fig. 6a and Additional file 1: Table S2). All of the OATPs were predicted to carry at least 2 myristoylation sites (Fig. 6b and Additional file 1: Table S2), where OATP from *An. gambiae* (XP_557860) was predicted to carry the highest number (24 sites) and *I. scapularis* OATPs (DAA34891, XP_002400770, XP_002404592, XP_002415171) were predicted to carry the lowest (3 sites) number of myristoylation sites.

**Fig. 7:**
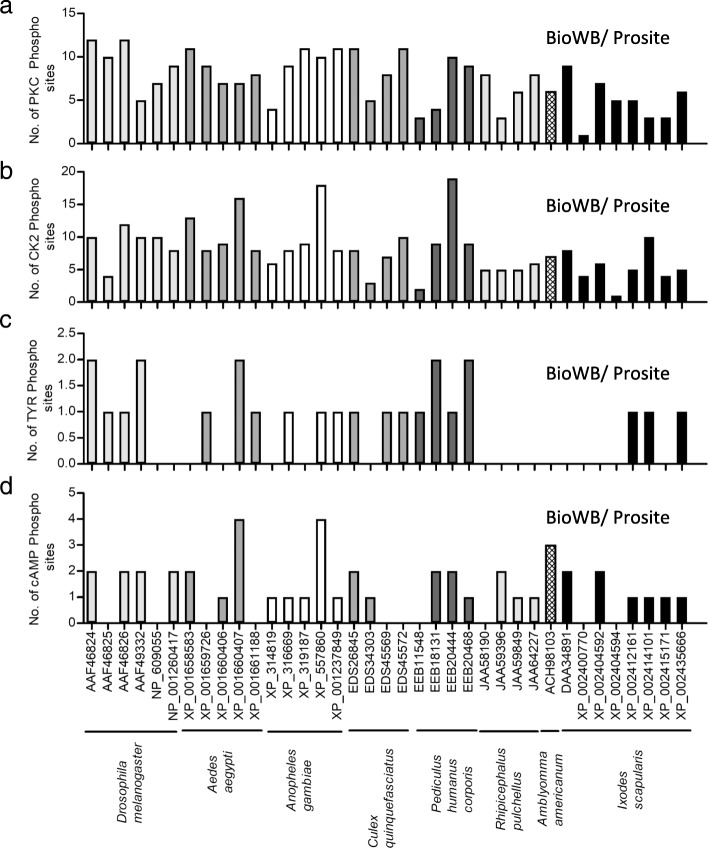
Analysis of phosphorylation sites in OATPs from medically important vectors. Amino acid sequences of several OATPs were individually analyzed at Biology WorkBench or PROSITE databases for PKC phosphorylation (**a**), CK2 phosphorylation (**b**), tyrosine phosphorylation (**c**) and cAMP-dependent phosphorylation sites (**d**). Histograms represent the number of post-translational modification sites for each OATP. The post-translational modifications in the transmembrane regions within each OATP were not considered for histogram plots. GenBank accession numbers and organism names are shown at the bottom of the figure

All 37 OATPs analyzed in this study were predicted to carry varying numbers of PKC phosphorylation sites (Fig. 7a and Additional file 1: Table S3). The *D. melanogaster* OATPs (AAF46824, AAF46826) were predicted to carry the highest number (12 sites) and *I. scapularis* OATP (XP_002400770) was predicted to carry the lowest number (1 site) of PKC phosphorylation sites (Fig. 7a and Additional file 1: Table S3). In addition, out of the 37 OATPs analyzed, all were predicted to carry at least one CK2 phosphorylation site (Fig. 7b and Additional file 1: Table S3). *P. humanus corporis* OATP (EEB20444) was predicted to carry the highest number (19 sites) and *I. scapularis* OATP (XP_002404594) was predicted to carry the lowest number (1 site) of CK2 phosphorylation sites (Fig. 7b and Additional file 1: Table S3). Tyrosine phosphorylation sites were also evident in 20 out of the 37 OATPs that were analyzed (Fig. 7c and Additional file 1: Table S3). All four sequences from *P. humanus corporis* OATPs, four out of six *D. melanogaster* sequences, three out of four *Cx. quinquefasciatus* sequences and three out of five *An. gambiae* sequences contained at least one tyrosine phosphorylation site (Fig. 7c). Interestingly, out of 13 OATP sequences from ticks, only three sequences from *I. scapularis* (XP_002412161, XP_002414101, XP_002435666) were predicted to carry tyrosine phosphorylation sites (Fig. 7c). Among all OATPs, *Ae. aegypti* OATP (XP_001660407), *P. humanus corporis* OATPs (EEB18131, EEB20468) and *D. melanogaster* OATPs (AAF46824, AAF49332) were predicted to carry the highest number (2 sites) of tyrosine phosphorylation sites (Fig. 7c and Additional file 1: Table S3). Out of 37 OATPs, 27 were predicted to carry at least one cAMP-dependent protein kinase phosphorylation site (Fig. 7d and Additional file 1: Table S3). The *Ae. aegypti* OATP (XP_001660407) and *An. gambiae* OATP (XP_557860) were predicted to carry the highest number (4 sites) of cAMP-dependent protein kinase phosphorylation sites (Fig. 7d and Additional file 1: Table S3). In addition, with the exception of three *I. scapularis* OATPs (XP_002400770, XP_002404594, XP_002415171) and one *D. melanogaster* OATP (AAF46825), all 33 other OATPs were predicted to carry one Kazal domain in their sequences (Additional file 1: Figure S3).

## Discussion

The development of a broad-spectrum anti-vector vaccine to control or treat diseases transmitted by ticks, mosquitoes and lice largely depends on the characterization of conserved proteins present in them. Our previous study provided evidence on the role of OATP family proteins in the survival of rickettsial pathogen in ticks [12]. In this study, we provide evidence on the role of OATP family proteins in the survival of intracellular tick-borne viruses. This study, in conjunction with our previous findings, clearly recognizes arthropod OATPs as molecular players targeted by vector-borne pathogens and in particular by intracellular pathogens for their survival in the vector host.

The observation of no difference in OATP gene expression between *B. burgdorferi* and LGTV-infected ticks in comparison to their uninfected controls in unfed tick developmental stage cannot rule out the possibility that these pathogens may impact *oatp* expression during initial phases of their infection in ticks. We used the ISE6 *in vitro* tick cell line and LGTV-infection model to address whether intracellular pathogens have any impact on *oatp* expression in the early part of their infection of tick cells. We considered 24 and 72 h, as early and late time points of infection, respectively. The significant increase in LGTV burden at 72 h pi in comparison to 24 h pi, and observation of significant downregulation of *oatps* at 24 h pi but not at 72 h pi, clearly suggests an initial vector-host response to control viral replication at an early infection phase. Our data suggest that OATPs may be critical for initial replication of LGTV and that the host is downregulating these arthropod molecules to control viral replication. The observation of a significant reduction in viral loads upon treatment of tick cells with OATP inhibitor supports this hypothesis.

The inhibitor SPZ is a general OATP inhibitor that could block the function of tick OATPs. In addition, SPZ is proposed to be a nonselective uridine 5'-diphospho-glucuronosyltransferase (UGT) inhibitor and a substrate for ATP-binding cassette (ABC) transporters such as multidrug resistance proteins MRP2 [39, 40]. The observation of differential modulation (up/downregulation) of OATP transcripts upon treatment of LGTV-infected tick cells with SPZ suggests that the transcriptional regulation of OATPs could be interdependent to each other. The blocking of one OATP may have an effect on the transcription of other OATPs. As SPZ is related to ABC-transporters or affect glucuronosyltransferases, the roles for these molecules in the regulation of OATPs or in LGTV-tick interactions cannot be excluded. In our previous study, we observed that knockdown of *kat* gene expression affected *isoatp4056* expression [12]. We proposed that xanthurenic acid (XA), a metabolite from tryptophan pathway and a product of KAT enzyme, is important in the regulation of *isoatp4056* expression. Consistent with our previous *A. phagocytophilum*-infection model [12], we noticed a low expression of *isoatp4056* and reduced levels of *kat* transcripts upon SPZ-treatment of LGTV-infected tick cells. Collectively, these observations suggest a highly interdependent pathway among OATPs with KAT.

In this study, putative post-translational modifications on various arthropod OATPs were predicted and analyzed. Glycosylation is an important post-translational modification that is commonly observed in membrane proteins [41]. A study has shown that glycosylation could impact membrane targeting and/or maintenance of protein stability [42]. Based on the presence of glycosylation sites on several OATPs analyzed in this study, post-translational modification might be essential for OATPs function at the host cell membrane during early phase of interactions with intracellular pathogens. As evidenced in our comparative analysis, *Ae. aegypti* OATP (XP_001660407) contains the highest number of glycosylation sites in comparison to the other OATPs from various arthropods. This study opens up an interesting question: does the level of glycosylation have any impact on the membrane targeting and/or maintenance of OATP protein stability during vector-pathogen interactions?

We previously demonstrated that a rickettsial pathogen modulates phosphorylation of actin in its vector host [43]. The observation of a higher number of CK2, PKC and cAMP phosphorylation sites in comparison to tyrosine phosphorylation sites suggests serine/threonine kinases are the important mediators of signaling in the medically important vectors. It was interesting to note that all but four of the OATPs carry one Kazal domain in their primary amino acid sequence (Additional file 1: Figure S3). Kazal domain containing serine proteases plays crucial roles in various physiological mechanisms in several organisms including arthropod blood-feeding [37, 44]. A study by Mulenga et al. [37] has shown that *A. americanum* ticks treated with OATP-dsRNA had lower engorgement weights in comparison to the control group, suggesting the importance of OATP in blood-feeding. The presence of the Kazal domain in most of the OATPs suggests that this domain could be an ideal target for the development of a broad-spectrum anti-vector vaccine against various arthropods.

## Conclusions

In conclusion, this study provides additional important evidence on the role of OATP in the interactions of vector with bacteria and viruses, in particular with intracellular pathogens. Studies such as this in understanding the roles of OATPs in vector-pathogen interactions might provide novel universal strategies to combat several tick-borne bacterial and/or viral diseases.

## Additional file

Additional file 1: **Figure S1.** LGTV infection has no impact on tick cell morphology. Representative phase contrast images of uninfected or LGTV-infected tick cells at 24 and 72 h pi. UI indicates uninfected and LGTV indicates LGTV-infected tick cells. *Scale-bars*: 200 μm. **Figure S2.** SPZ treatment has no cytotoxic effects on tick cells. Representative phase contrast images of untreated or mock- or 100 μM of SPZ uninfected tick cells at 4, 24, 48 and 72 h post-treatment (pt). *Scale-bars*: 200 μm. **Figure S3.** Analysis of Kazal domain sites in OATPs from medically important vectors. Amino acid sequences of OATPs were individually analyzed at NCBI conserved domain search and PROSITE databases for prediction of Kazal domain sites in several OATPs. Histograms represent number of KAZAL sites for each OATP. Full-length OATP sequences were considered to determine KAZAL sites. GenBank accession numbers and organism names are shown at the bottom of the figure. **Table S1.** Summarizing statistical test outcomes for the data in this paper. **Table S2.** The numbers of ASN glycosylation and myristoylation sites predicted from either outside (external) or inside (internal) regions of OATPs but not in the transmembrane regions are shown. Aa indicates total number of amino acids and TM indicates number of transmembrane regions. **Table S3.** Summarizing number of phosphorylation sites in different OATPs. The numbers of cAMP, PKC, CK2 and tyrosine phosphorylation sites (predicted either outside or inside regions of OATPs but not in the transmembrane regions) are shown. Aa indicates total number of amino acids, TM indicates number of transmembrane regions, Int. indicates number of sites in the inside region and Ext. indicates number of sites on the outside regions of OATPs. (PDF 12295 kb)

## Abbreviations

ASN: Asparagine N-glycosylation
CK2: Casein kinase II
KAT: Kynurenine aminotransferase
LGTV: Langat virus
OATPs: Organic anion transporting polypeptides
pi: Post-infection
PKC: Protein kinase C
QRT-PCR: Quantitative real-time polymerase chain reaction
SPZ: ±-sulfinpyrazone
TBEV: Tick-borne encephalitis virus
XA: Xanthurenic acid
