# Supplementary material for: Characterization of tick organic anion transporting polypeptides (OATPs) upon bacterial and viral infections
Source: Parasit Vectors. 2018 Nov 14;11:593. doi: 10.1186/s13071-018-3160-6 (PMC6236954; doi:10.1186/s13071-018-3160-6)
Supplement: Supplementary file 1 — Figure S1. LGTV infection has no impact on tick cell morphology. Representative phase contrast images of uninfected or LGTV-infected tick cells at 24 and 72 h pi. UI indicates uninfected and LGTV indicates LGTV-infected tick cells. Scale-bars: 200 μm. Figure S2. SPZ treatment has no cytotoxic effects on tick cells. Representative phase contrast images of untreated or mock- or 100 μM of SPZ uninfected tick cells at 4, 24, 48 and 72 h post-treatment (pt). Scale-bars: 200 μm. Figure S3. Analysis of Kazal domain sites in OATPs from medically important vectors. Amino acid sequences of OATPs were individually analyzed at NCBI conserved domain search and PROSITE databases for prediction of Kazal domain sites in several OATPs. Histograms represent number of KAZAL sites for each OATP. Full-length OATP sequences were considered to determine KAZAL sites. GenBank accession numbers and organism names are shown at the bottom of the figure. Table S1. Summarizing statistical test outcomes for the data in this paper. Table S2. The numbers of ASN glycosylation and myristoylation sites predicted from either outside (external) or inside (internal) regions of OATPs but not in the transmembrane regions are shown. Aa indicates total number of amino acids and TM indicates number of transmembrane regions. Table S3. Summarizing number of phosphorylation sites in different OATPs. The numbers of cAMP, PKC, CK2 and tyrosine phosphorylation sites (predicted either outside or inside regions of OATPs but not in the transmembrane regions) are shown. Aa indicates total number of amino acids, TM indicates number of transmembrane regions, Int. indicates number of sites in the inside region and Ext. indicates number of sites on the outside regions of OATPs. (PDF 12295 kb) [file 13071_2018_3160_MOESM1_ESM.pdf]

Characterization of tick organic anion transporting polypeptides (OATPs) upon bacterial and viral infections

Vikas Taank [1], Wenshuo Zhou [1], Xuran Zhuang [2], John F. Anderson [3], Utpal Pal [2], Hameeda Sultana [1, 4] and Girish Neelakanta [1, 4, *]

Additional file 1

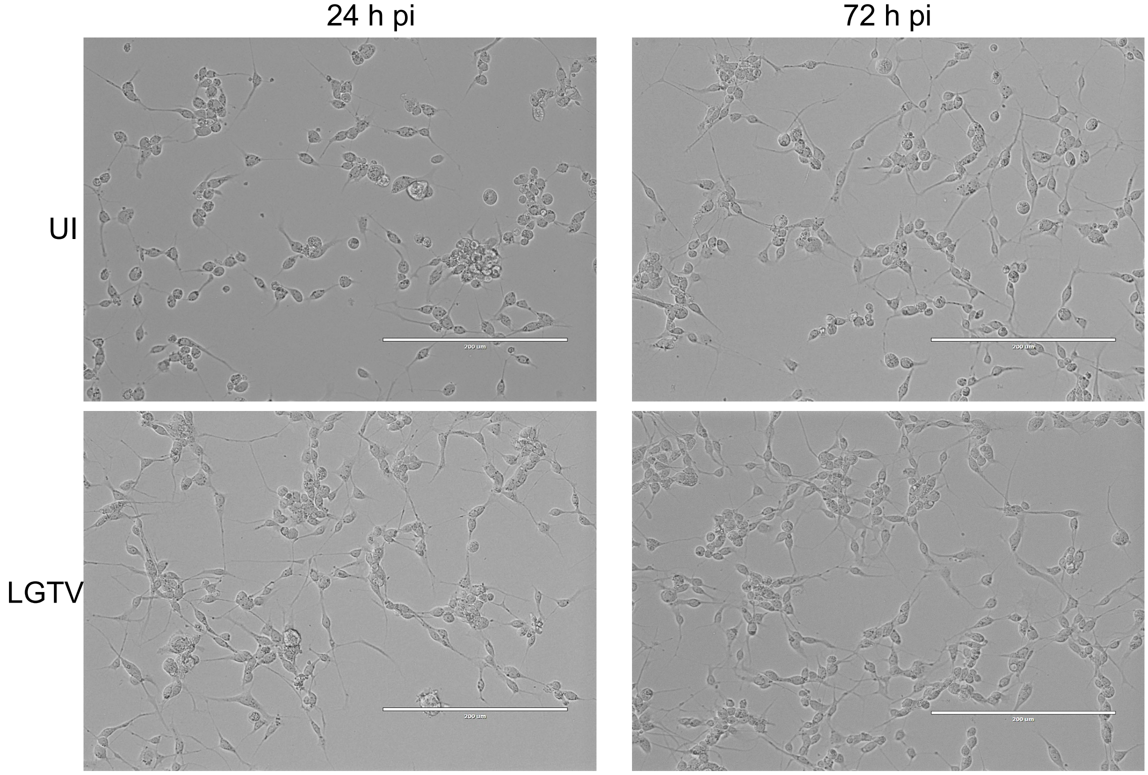

Figure S1. LGTV infection has no impact on tick cell morphology. A) Representative phase contrast images of uninfected or LGTV-infected tick cells at 24 and 72 h pi. UI indicates uninfected and LGTV indicates LGTV-infected tick cells. Scale bars: 200 µm

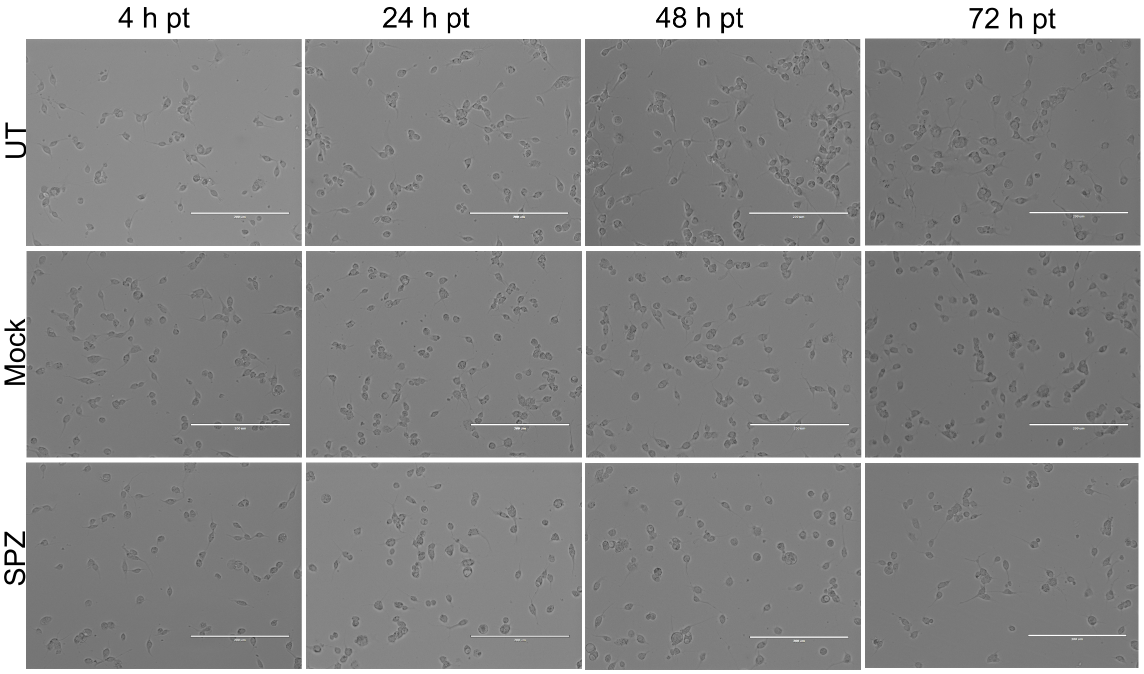

Figure S2. SPZ treatment has no cytotoxic effects on tick cells. Representative phase contrast images of untreated or mock- or 100 μM of SPZ uninfected tick cells at 4, 24, 48 and 72 h post treatment (pt) are shown. Scale bars: 200 μm

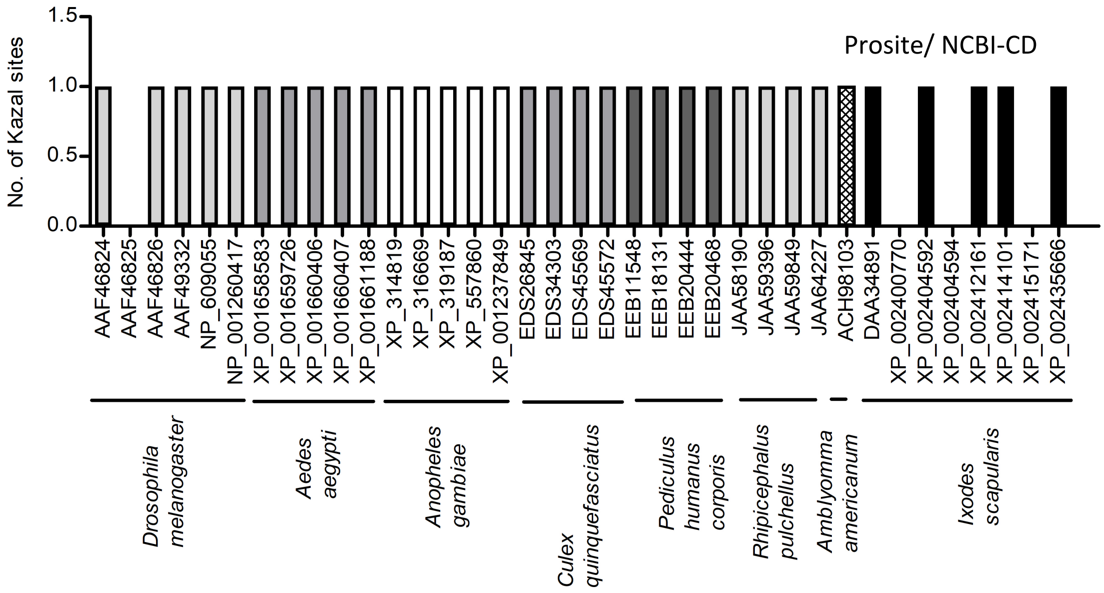

Figure S3. Analysis of Kazal domain sites in OATPs from medically important vectors. Amino acid sequences of OATPs were individually analyzed at NCBI conserved domain search and PROSITE databases for prediction of Kazal domain sites in several OATPs. Histograms represent number of KAZAL sites for each OATP. Full-length OATP sequences were considered to determine KAZAL sites. GenBank accession numbers and organism names are shown at the bottom of the figure.

Table S1: Summarizing statistical test outcomes for the data in this paper.

Figure	*Gene*	Samples	Student's t test (P value)	Mann Whitney test (P value)
1 A	*isoatp0726*	Uninfected, *Borrelia burgdorferi* infected	0.4881	0.7959
1 B	*isoatp2114*		0.3707	0.4359
1 C	*isoatp2116*		0.8931	0.2428
1 D	*isoatp4056*		0.5938	0.0753
1 E	*isoatp4134*		0.6086	1.0000
1 F	*isoatp4548*		0.4358	0.2799
1 G	*isoatp4550*		0.5152	0.4967
1 H	*isoatp5126*		0.1765	0.7959
1 I	*isoatp5621*		0.9685	0.7959
1 J	*kat*		0.0571	0.0605
1 K	*FlaB*		**0.0175**	**< 0.0001**
2 A	*isoatp0726*	Uninfected, LGTV infected ticks	0.8088	0.7959
2 B	*isoatp2114*		0.5110	0.8534
2 C	*isoatp2116*		0.8414	0.4813
2 D	*isoatp4056*		0.2193	0.5288
2 E	*isoatp4134*		0.1787	0.6038
2 F	*isoatp4548*		0.4263	0.9188
2 G	*isoatp4550*		0.7449	1.0000
2 H	*isoatp5126*		0.1912	0.3562
2 I	*isoatp5621*		0.6316	0.3930
2 J	*kat*		0.2064	0.2756
2 K	LGTV +		**< 0.0001**	**< 0.0001**
2 L	LGTV -		**< 0.0001**	**< 0.0001**
3 A	*isoatp0726*	24 h, Uninfected, LGTV infected tick cells	0.1813	0.1797
3 B	*isoatp2114*		**0.0010**	**0.0022**
3 C	*isoatp2116*		0.1255	0.1320
3 D	*isoatp4056*		0.0960	0.0931
3 E	*isoatp4134*		0.1180	0.1320
3 F	*isoatp4548*		**0.0059**	**0.0152**
3 G	*isoatp4550*		**0.0325**	**0.0411**
3 H	*isoatp5126*		**0.0191**	**0.0152**
3 I	*isoatp5621*		0.5253	0.8182
3 J	*kat*		0.5463	0.8182
3 K	LGTV +		**< 0.0001**	**0.0022**
3 A	*isoatp0726*	72 h, Uninfected, LGTV infected tick cells	0.0767	0.0931
3 B	*isoatp2114*		0.6696	0.6991
3 C	*isoatp2116*		0.0946	0.1320
3 D	*isoatp4056*		0.0963	0.0931
3 E	*isoatp4134*		0.2200	0.2403
3 F	*isoatp4548*		0.2021	0.1255
3 G	*isoatp4550*		0.1999	0.3939
3 H	*isoatp5126*		0.2964	0.6991
3 I	*isoatp5621*		0.2257	0.9372
3 J	*kat*		0.9894	0.6991
3 K	LGTV +		**< 0.0001**	**0.0022**
4 B	LGTV	Mock, SPZ treatment	**0.0252**	**0.0418**
5 A	*isoatp0726*	Mock, SPZ treatment	**0.0216**	**0.0350**
5 B	*isoatp2114*		0.2141	0.2224
5 C	*isoatp2116*		0.3904	0.3527
5 D	*isoatp4056*		**0.0024**	**0.0006**
5 E	*isoatp4134*		**0.0159**	**0.0279**
5 F	*isoatp4548*		0.6194	0.4967
5 G	*isoatp4550*		**0.0259**	**< 0.0001**
5 H	*isoatp5126*		0.8399	0.1655
5 I	*isoatp5621*		**0.0311**	**0.0279**
5 J	*kat*		**0.0151**	**0.0001**

Table S2: Summarizing number of glycosylation and myristoylation sites in different OATPs. The numbers of ASN glycosylation and myristoylation sites predicted from either outside (external) or inside (internal) regions of OATPs but not in the transmembrane regions are shown. Aa indicates total number of amino acids and TM indicates number of transmembrane regions.

Organism	Accession	Aa	TM	Oatp Motif	ASN Glycosylation sites			Myristoylation sites		
				(WxGxWWxG)	Internal	External	Total	Internal	External	Total
Aedes aegypti	XP_001658583	815	12	+	0	5	5	1	3	4
	XP_001659726	664	12	+	1	4	5	2	2	4
	XP_001660406	752	11	+	5	4	9	2	6	8
	XP_001660407	1266	11	+	6	8	14	5	16	21
	XP_001661188	728	12	+	0	5	5	4	6	10
Anopheles gambiae	XP_314819	650	10	+	5	1	6	5	3	8
	XP_316669	772	12	+	0	4	4	3	4	7
	XP_319187	822	12	+	2	2	4	2	6	8
	XP_557860	1301	13	+	6	4	10	4	20	24
	XP_001237849	687	12	+	2	4	6	4	3	7
Culex quinquefasciatus	EDS_26845	821	12	+	0	2	2	2	2	4
	EDS_34303	629	9	+	4	3	7	1	3	4
	EDS_45569	708	12	+	1	5	6	1	6	7
	EDS_45572	764	11	+	0	4	4	3	3	6
Pediculus humanus corporis	EEB_11548	607	11	+	0	6	6	2	3	5
	EEB_18131	751	12	+	2	7	9	0	5	5
	EEB_20444	996	11	+	1	7	8	2	5	7
	EEB_20468	864	12	+	2	8	10	2	6	8
Rhipicephalus pulchellus	JAA_58190	748	12	+	1	3	4	5	3	8
	JAA_59396	585	10	+	0	2	2	1	5	6
	JAA_59849	798	12	+	0	4	4	10	4	14
	JAA_64227	734	11	+	3	0	3	4	5	9
Amblyomma americanum	ACH_98103	733	12	+	1	1	2	2	5	7
Ixodes scapularis	DAA_34891	668	12	+	2	1	3	1	2	3
	XP_002400770	590	12	+	0	3	3	1	2	3
	XP_002404592	528	10	+	2	1	3	1	2	3
	XP_002404594	290	6	+	0	0	0	2	2	4
	XP_002412161	576	11	+	1	3	4	3	5	8
	XP_002414101	701	7	+	5	0	5	3	4	7
	XP_002415171	257	5	+	0	0	0	0	3	3
	XP_002435666	648	12	+	1	1	2	1	7	8
Drosophila melanogaster	AAF46824	684	12	+	2	2	4	1	2	3
	AAF46825	680	12	+	1	4	5	1	4	5
	AAF46826	789	12	+	3	6	9	1	5	6
	AAF49332	819	12	+	2	6	8	9	4	13
	NP_609055	692	12	+	4	2	6	4	4	8
	NP_001260417	745	11	+	2	6	8	2	3	5

Table S3: Summarizing number of phosphorylation sites in different OATPs. The numbers of cAMP, PKC, CK2 and tyrosine phosphorylation sites (predicted either outside or inside regions of OATPs but not in the transmembrane regions) are shown. Aa indicates total number of amino acids, TM indicates number of transmembrane regions, Int. indicates number of sites in the inside region and Ext. indicates number of sites on the outside regions of OATPs.

Organism	Accession	Aa	TM	OATP Motif (WxGxW WxG)	CAMP_PHOSPHO			PKC_PHOSPHO			CK2_PHOSPHO			TYR_PHOSPHO		
					Int.	Ext.	Total	Int.	Ext.	Total	Int.	Ext.	Total	Int.	Ext.	Total
Aedes aegypti	XP_001658583	815	12	+	2	0	2	4	7	11	3	10	13	0	0	0
	XP_001659726	664	12	+	0	0	0	5	4	9	2	6	8	0	1	1
	XP_001660406	752	11	+	0	1	1	0	7	7	4	5	9	0	0	0
	XP_001660407	1266	11	+	1	3	4	3	4	7	7	9	16	2	0	2
	XP_001661188	728	12	+	0	0	0	6	2	8	6	2	8	0	1	1
Anopheles gambiae	XP_314819	650	10	+	0	1	1	1	3	4	5	1	6	0	0	0
	XP_316669	772	12	+	1	0	1	6	3	9	4	4	8	0	1	1
	XP_319187	822	12	+	1	0	1	5	6	11	3	6	9	0	0	0
	XP_557860	1301	13	+	1	3	4	2	8	10	5	13	18	1	0	1
	XP_001237849	687	12	+	1	0	1	9	2	11	5	3	8	0	1	1
Culex quinquefasciatus	EDS_26845	821	12	+	2	0	2	6	5	11	2	6	8	0	1	1
	EDS_34303	629	9	+	1	0	1	2	3	5	3	0	3	0	0	0
	EDS_45569	708	12	+	0	0	0	3	5	8	3	4	7	0	1	1
	EDS_45572	764	11	+	0	0	0	6	5	11	2	8	10	0	1	1
Pediculus humanus corporis	EEB_11548	607	11	+	0	0	0	2	1	3	1	1	2	0	1	1
	EEB_18131	751	12	+	1	1	2	2	2	4	4	5	9	0	2	2
	EEB_20444	996	11	+	2	0	2	5	5	10	7	12	19	0	1	1
	EEB_20468	864	12	+	1	0	1	6	3	9	5	4	9	1	1	2
Rhipicephalus pulchellus	JAA_58190	748	12	+	0	0	0	7	1	8	2	3	5	0	0	0
	JAA_59396	585	10	+	1	1	2	1	2	3	2	3	5	0	0	0
	JAA_59849	798	12	+	1	0	1	4	2	6	1	4	5	0	0	0
	JAA_64227	734	11	+	1	0	1	6	2	8	3	3	6	0	0	0
Amblyomma americanum	ACH_98103	733	12	+	2	1	3	4	2	6	4	3	7	0	0	0
Ixodes scapularis	DAA_34891	668	12	+	1	1	2	5	4	9	4	4	8	0	0	0
	XP_002400770	590	12	+	0	0	0	1	0	1	1	3	4	0	0	0
	XP_002404592	528	10	+	2	0	2	3	4	7	4	2	6	0	0	0
	XP_002404594	290	6	+	0	0	0	3	2	5	1	0	1	0	0	0
	XP_002412161	576	11	+	1	0	1	2	3	5	2	3	5	0	1	1
	XP_002414101	701	7	+	1	0	1	1	2	3	7	3	10	1	0	1
	XP_002415171	257	5	+	1	0	1	3	0	3	2	2	4	0	0	0
	XP_002435666	648	12	+	1	0	1	3	3	6	4	1	5	1	0	1
Drosophila melanogaster	AAF46824	684	12	+	2	0	2	7	5	12	6	4	10	1	1	2
	AAF46825	680	12	+	0	0	0	5	5	10	2	2	4	0	1	1
	AAF46826	789	12	+	1	1	2	9	3	12	9	3	12	0	1	1
	AAF49332	819	12	+	2	0	2	3	2	5	6	4	10	2	0	2
	NP_609055	692	12	+	0	0	0	2	5	7	5	5	10	0	0	0
	NP_001260417	745	11	+	1	1	2	4	5	9	2	6	8	0	0	0
